# 

**Published:** 1914-10-10

**Authors:** 


### CONTENTS.

| | PAGE |
|---|---|
| **Journalism and the Hospitals** ... ... ... | 23 |
| **A Foreword to Fellow-Workers** ... ... ... | 24 |
| **Hospital and Institutional News** ... ... ... | 25 |

The Hospital's Special Number—Technical Equipment Means Joy for the Workers—Why The Hospital's Readers are Diligent Readers—Matters of Special Interest—Hospital Gardens—The Equipment of Territorial Hospitals—The Voluntary Hospitals and the War—The Welsh Hospital—A Working-class Cot in Manchester—Medical Officers and the War—The Red Cross on Annual Reports—Surgeons for Serbia—Mental Hospitals' Staffs and Recruiting—From Chaplain to Chairman at Portsmouth—Block Floor Replaced by Linoleum—Conducting an Appeal While on Active Service—Position of the Hospital Saturday Fund—Panel Doctor's Attempt to Charge an Insured Patient—A Great Scotch Practitioner—The Bath Statutory Hospital Extension — The Rubber Floor at Guy's Hospital—Dublin Hospitals and the Insurance Act—The Suggested Remedy of Grants-in-Aid—The South Manchester Children's Hospital—Bradford, the New Infirmary—A Children's Friend—The War and Mental Disease—Tuberculosis Hospitals for the Wounded—This Week's Drug Market—To Our Readers.

| | PAGE |
|---|---|
| **What a Storehouse is the Voluntary Hospital!** | 31 |
| **Some Workers of Note** ... ... ... ... | 32 |
| Louisa Warnes, 1836-1914. | |
| **Territorial Hospitals** ... ... ... ... ... | 33 |
| The Guiding Principles of the Scheme. | |
| The Fifth Northern General Hospital, Leicester. | |
| **Buildings Contemplated** ... ... ... ... | 37 |
| **The Territorial Hospital, Leicester** ... ... | 38 |
| **The Growth of Hospital Literature** ... ... | 40 |
| The New Tendency and its Significance. | |
| **Hospitals and the War** ... ... ... ... | 41 |
| **The Work of London and Provincial Hospitals** | |
| Hampstead General Hospital—The Women's War Hospital. | |

[*See over.*]

### CONTENTS—(continued).

PAGE

**Soldiers and the Provincial Hospitals**
Bath—Bristol : Arrival of Serious Cases—Cambridge : Unloading a Hospital Train—Cardiff : A Wounded Soldier's Thanks—Chatham District : King and Queen's Visit—Gravesend and District : Soldiers' Tales—Leicester : A Third Batch of Wounded—Sheffield : The Third Convoy—The First Wounded at Tunbridge Wells.

**The Southern Territorial Hospital, Portsmouth**

**Hospital Economics** ... ... ... ... ... ... 45
King Edward's Hospital Fund for London Statistics (First Notice).

**Round the World** ... ... ... ... ... ... 46

Fellow-Workers and the Roll of Honour.

**Reports on Hospitals of the United Kingdom**— 47
By SIR HENRY BURDETT, K.C.B., K.C.V.O.
The Cumberland Infirmary, Carlisle.
Imperfections in the Cumberland Infirmary.

**Some Home-Made Hospital Equipment...** ... 49
Examples from Leicester.

**An Irish Hospital Train** ... ... ... ... ... 51

**The Motor-Cycle Ambulance** ... ... ... 51

**Institutional Needs** ... ... ... ... ... ... 52

